# Quality of Life in Liver Transplant Recipients: A Retrospective Study

**DOI:** 10.3390/ijerph17113809

**Published:** 2020-05-27

**Authors:** Rosario Girgenti, Alessandro Tropea, Maria Antonina Buttafarro, Rosalia Ragusa, Martina Ammirata

**Affiliations:** 1 Clinical Psychology Service, IRCCS-ISMETT (Istituto Mediterraneo per i Trapianti e Terapie ad Alta Specializzazione), 90127 Palermo, Italy; rgirgenti@ismett.edu (R.G.); mbuttafarro@ismett.edu (M.A.B.); martina.ammirata@libero.it (M.A.); 2 Department for the Treatment and Study of Abdominal Diseases and Abdominal Transplantation, IRCCS-ISMETT (Istituto Mediterraneo per i Trapianti e Terapie ad Alta Specializzazione), 90127 Palermo, Italy; ragusar@unict.it; 3 Health Technology Assessment Committee, University Hospital “G. Rodolico”, 95100 Catania, Italy

**Keywords:** quality of life, liver transplantation, compliance, surgery

## Abstract

Background: This study aims to investigate the quality of life and the therapeutic compliance of the patients who received a liver transplant, from a living or deceased donor, at IRCCS ISMETT in the last three years. Liver transplantation is an effective therapeutic strategy for patients with end-stage liver failure. The quality of life (QOL) of liver disease patients is placed under considerable stress due to the debilitating clinical conditions and related issues experienced in everyday life by these individuals. The concept of QOL is being increasingly used to define the individual perception of health, including physical, mental, and social wellbeing. The success of a liver transplant should therefore be intended not only in terms of survival, but also of recovery of a satisfying quality of life. For this reason, our liver transplant recipients are closely monitored and supported from a psychological standpoint. This is done to monitor their ability to adapt to and comply with their clinical condition and to verify their gradual resumption of their path of life Methods: We retrospectively analyzed data collected by the IRCCS ISMETT Clinical Psychology Service during routine psychological follow-up of liver transplant recipients. Data refer to 82 patients who received a liver transplant between January 2017 and September 2019 and describe their QOL and therapeutic adherence. The obtained results were compared with the main studies on this issue available in literature. Results: Ninety-four percent of liver transplant recipients reported high mean scores of quality of life and therapeutic adherence 28% of patients reported at least one persistent annoying symptom after transplantation, although in some cases this did not affect the overall QOL. The results also refer to patients with a pre-transplant diagnosis of alcohol-related cirrhosis, who confirm their complete abstinence from alcohol. Conclusions: Our results confirm the efficacy of the liver transplantation to achieve of a good QOL. Furthermore, these patients seem to maintain high therapeutic adherence, thus ensuring a good outcome of the care received during the transplantation process.

## 1. Introduction

In the last few years, the multiple medical progresses and improved surgical techniques have been allowed to significantly increase the number of solid organ transplants. Namely, liver transplantation is considered an effective therapeutic strategy for patients with end-stage liver failure, allowing them to survive and resume a satisfying quality of life. Following a period of rehabilitation that can vary in terms of time and potential post-surgery complications, the patient’s clinical conditions progressively improve and he/she is able to resume social and working tasks previously affected by the onset of the disease.

The recovery of the clinical performance status after the intervention is observed in several studies [1,2,3,4] and this gives hope of a better and longer life for these patients [5,6,7].

Clinical improvement positively impacts many aspects of daily life including overall health, physical performance, pain, quality of sleep, occupational status, vitality, social activity, and personal support. The improvements seem to be independent of the type of graft and of the time elapsed from surgery.

Achieving an adequate quality of life (QOL) is an indicator of therapeutic success that is becoming more and more important to measure by the patient care team after the transplant [8]. The World Health Organization defines QOL as individuals’ perception of their position in life in the context of the culture and value systems in which they live, and in relation to their goals, expectations, standards and concerns [9,10]. It is a broad ranging concept affected in a complex way by the person’s physical health, psychological state, level of independence, social relationships, personal beliefs, and their relationship to salient features of their life environment. Today the goal in all the fields of medicine is to guarantee an adequate QOL for the patient: physical and mental health are considered indicators of individual general wellbeing [11,12,13,14,15,16].

In the light of the above, it is important to know how our patients feel after the transplant, from a physical, psychological, existential, and relational standpoint [17,18,19,20,21,22,23,24]. The main goal of this study is to investigate the QOL of the patients who received a liver transplant, from a living or deceased donor, at IRCCS ISMETT in the last three years. The second goal is to monitor therapeutic compliance, considered a key aspect for a positive outcome of the transplant, and maintain the graft health in the long term.

## 2. Materials and Methods

### 2.1. Patients

Data of a sample of 82 patients (20 women and 62 men), aged 29–71, who underwent a liver transplant at IRCCS ISMETT between 2017 and 2019, were retrospectively analyzed. Data were collected during routine psychological follow-up performed by the IRCCS ISMETT Clinical Psychology Service and entered in a database that includes clinical and psychological information of all organ transplant recipients. In the light of the most modern surgical and post-surgical procedures, we selected adult patients who underwent a deceased-donor liver transplant in the last three years, with Italian nationality and living in Italy. We included in the study all adult liver transplant recipients who accepted to undergo an interview with the clinical psychologist. Patients with a diagnosis of double transplant and under the age of 18 years were excluded from this study. Clinical and demographic characteristics are summarized in Table 1.

### 2.2. Psychology Pathway for Transplant Candidates

**Figure 1:**
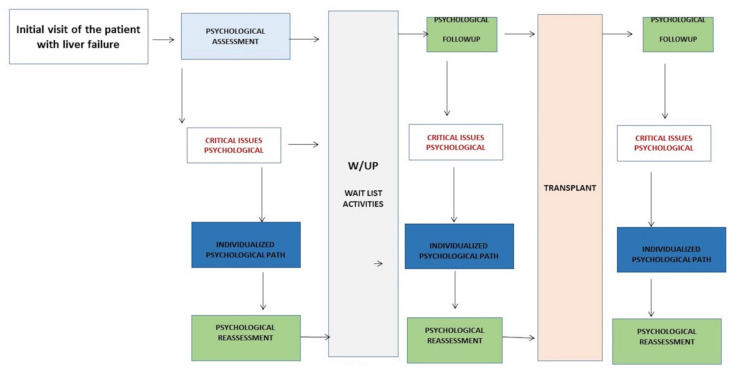
Summary scheme of taking charge of the patient by the ISMETT Clinical Psychology Unit. The scheme visualized the psychological assessment of the patient with liver disease in ISMETT from the first visit to the transplant.

The patient with an end-stage liver failure and an indication to liver transplantation was preliminary assessed by the Clinical Psychology Service that performs an accurate psychological evaluation and defines his or her eligibility to transplant (Figure 1).

A dedicated psychology pathway is designed for every single patient. The goal is to reduce potential psychological and emotional barriers and enhance personal and relational resources. The pathway is personalized for each patient and it involves the family and social system. It is shared with the multidisciplinary team and readjusted accordingly during the plan of care, before and after the transplant. A similar approach to the transplant recipient proved effective, as our data showed.

### 2.3. Tools

#### 2.3.1. Quality of Life

The McGill Quality of Life Questionnaire© (MQOL) [25] was used to assess the QOL. Its 16 items were divided in 5 scales: physical well-being, physical symptoms, psychological symptoms, existential well-being, and support. It also provides an overall score of the QOL perceived by the patient. Scores for each single item are grouped according to this scheme:

Physical well-being (0–10): the higher the score, the better the perceived physical well-being.

Physical symptoms (0–30): the higher the score, the lower the intensity of the physical symptoms.

Psychological symptoms (0–40): the higher the score, the lower the intensity of the psychological symptoms.

Existential well-being (0–60): the higher the score, the better the perceived existential well-being.

Support (0–20): the higher the score, the better the perceived support.

#### 2.3.2. Therapeutic Adherence

Therapeutic adherence was assessed administering the Morisky Medication Adherence Scale (MMAS) [26], a self-report questionnaire based on four simple questions with the following possible answers: YES (0)–NO (1). The total score results in the following categories: **high** (4), **medium** (2–3), and **low** (0–1) therapeutic adherence. The 4-item Morisky Green and Levine Adherence Scale (MMAS-8) is a structured self-reported medication adherence measure expanded from the widely used adherence, resulting in better psychometric properties (Cronbach’s alpha of 0.83 vs. 0.61). The scale was originally developed to measure medication adherence in hypertension but has since been used for measuring medication adherence in multiple chronic conditions, with good predictive sensitivity and validity [26,27].

### 2.4. Statistical Analysis

Data on QOL and therapeutic compliance of transplant recipients were reviewed to correct potential data entry errors, missing data and/or double answers, and subject to descriptive statistics (percentage, mean, and median). No correlations were performed.

## 3. Results

### 3.1. Analysis of Sociodemographic and Clinical Factors

This study included 82 patients (20 women and 62 men) aged 29–71. The average age of patients was 56 years. All patients in the sample underwent a liver transplant at IRCCS ISMETT between 2017 and 2019. On average, the patients had to wait 130 days before undergoing the transplant.

**Figure 2:**
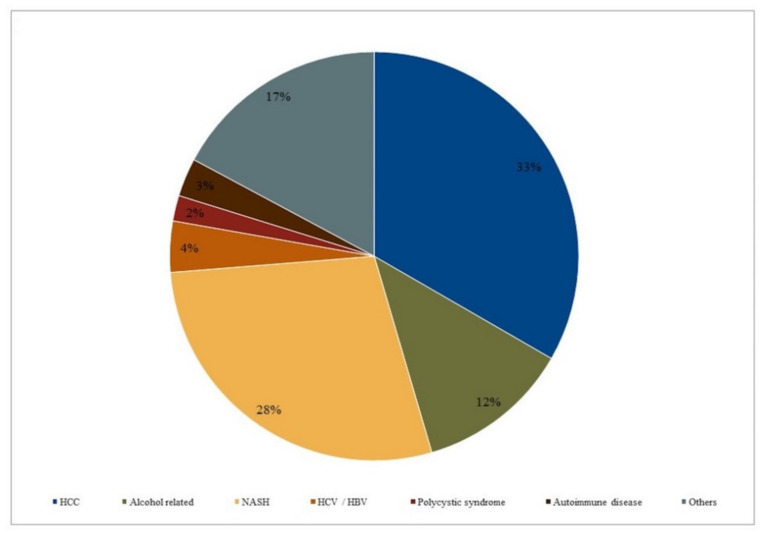
Main etiologies in the sample.

Most of the transplants were performed after a diagnosis of hepatocarcinoma (HCC). Twelve patients were diagnosed an alcohol-related cirrhosis, 7 a NASH-related cirrhosis, 4 alcohol- and HCV-related cirrhosis, 3 fulminant hepatitis, 2 polycystic syndrome, 2 HBV, and 3 autoimmune cirrhosis. The main etiologies in the sample are summarized in Figure 2.

### 3.2. Quality of Life

QOL results measured with the MQOL are reported in Table 2. Arithmetic mean values were obtained of the 5 summary scores deriving from the test scoring procedures, and a comparison made based on the time elapsed from the transplant.

Mean values of total QOL and physical/existential well-being were high. In order to compare the different scales, patients were divided between transplant recipients since less than two years, and recipients since more than two years. We witnessed a slight difference in the average scales of psychological symptoms between women and men: women presented lower levels of psychological well-being (23/40) than men (30/40). As for physical symptoms, 28% of patients reported at least one persisting annoying symptom after the transplant, namely the onset of abdominal hernia (Table 3).

### 3.3. Compliance

Following the MMAS scoring the arithmetic mean of the general therapeutic compliance in the total sample was obtained. Patients presented high therapeutic adherence (mean MMAS scale: 3.8/4).

### 3.4. Alcohol-Related Cirrhosis

**Figure 3:**
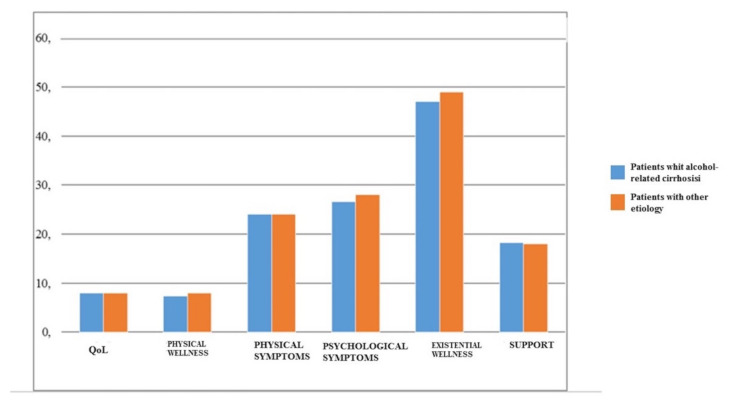
Comparison between patients transplanted for alcohol-related cirrhosis and patients with 173 other etiologies.

In the light of the obtained data, we particularly focused on transplant recipients with a history of alcoholism. Patients in the study transplanted for alcohol-related cirrhosis reported similar QOL levels compared to the sample with different diagnosis, according to MQOL (Figure 3) and maintained a high therapeutic adherence (mean MMAS scale: 4/4). All interviewed patients with a pre-transplant diagnosis of alcohol-related liver disease reported ongoing abstention from alcohol and confirmed their willingness to collaborate as they became aware of their previous dysfunctional conduct and its impact on their health.

In the questionnaire many patients described the transplant as a new birth and turning point for their life. What follows are some of the patients’ recurrent and significant comments:

“I’ve forgotten about alcohol. I’m no longer that person anymore”.

“I almost died and then came back to life again”.

“For me the transplant was like being born again, a new possibility. I’m not going to make those same mistakes again”.

## 4. Discussion

The obtained results confirmed the need of a close clinical and psychological monitoring for liver transplant recipients.

Ninety-four percent of the interviewed liver recipients reported an excellent overall quality of life and a MQOL score higher than 7. Although a large number of them reported residual physical symptoms, mainly due to surgical sequelae (8.5% reported developing abdominal hernia after the transplant), these symptoms do not seem to have affected the perceived physical and existential well-being, which remained high. Additionally, the perceived relational support was satisfying, and the reported therapeutic adherence was classified as “high” according to MMAS.

These results confirmed the significant benefits perceived by transplant recipients in their overall QOL, consistently with the main studies published in literature on the QOL after a liver transplant (Table 4).

Such a perception also seems to positively impact on adapting to the condition of transplant recipient and enhancing the tolerance of residual more or less life-limiting physical symptoms, if any.

The psychological symptoms described by the interviewed patients do not seem to limit particularly their psychological and clinical recovery. Anxiety and depression seem to be more significantly present in women than men, as also confirmed by published literature [2]. The presence of these symptoms seems to have no repercussions on the global existential well-being, which remains satisfactory in both men and woman.

The interviewed patients appeared to greatly appreciate the post-transplant physical and psychological improvements. This confirms the importance of the transplant for the patients’ attitude: they assign a new and positive meaning to life after transplant [13], acknowledge the post-transplant benefits, and give little or no importance to unforeseen clinical events.

Nevertheless, 18% of the interviewed sample required a customized psychological plan in the post-transplant period. This is because transplantation is a traumatic event that needs to be processed, even after many years.

Patients with a low QOL reported fatigue, slowing pace, and physical issues (Table 3) preventing them from resuming their normal everyday tasks.

Patients who reported an unsatisfactory QOL are followed by the Clinical Psychology Service with customized therapeutic programs, as they showed symptoms such as anxiety and depression in their post-transplant phase, as well as difficulties adapting to their new clinical condition. This suggests that not accepting the experience of the illness and failing to process their new clinical condition causes patients to badly adapt to their status of transplant recipients, decreasing their ability to tolerate physical symptoms and post-transplant complications.

The Clinical Psychology Service particularly focuses on transplant recipients for alcohol-related cirrhosis. The goal with these patients is to assess a full recovery of their QOL and also to verify that they maintain total abstinence from alcohol in order to prevent relapses to earlier patterns of dysfunctional behavior. Recent studies on alcohol relapse after liver transplantation confirm the need and value of a constant psychotherapy program during the first years after the transplant [28,29].

Transplant recipients for alcohol-related cirrhosis showed similar QOL levels compared to the group sample with different diagnoses, according to the MQOL (Figure 3) and maintained a high therapeutic adherence (mean Morisky scale: 4/4). Furthermore, the interviewed patients reported no episodes of alcohol relapse or craving after the transplant. This confirms the results in literature on the efficacy of psychology programs for alcohol-related cirrhosis patients. These programs should include an accurate pre-transplant assessment, continuing pre- and post-transplant monitoring, and customized psychotherapy programs to enhance the patient’s personal and family resources, and reduce the risk of relapses after liver transplantation [30,31]. This subject does however require additional studies to solve the controversy that still surrounds liver transplant selection criteria for alcohol-related cirrhosis patients [32,33].

Finally, the data confirm the need for a close psychological post-transplant follow-up for all patients, to monitor their gradual and satisfactory QOL recovery and early detection of any emotional discomfort that may prevent their full psychophysical recovery deteriorating any psychopathological dysfunctions.

## 5. Conclusions

The aim of this study was to describe the QOL of patients who underwent a liver transplant at IRCCS ISMETT over the last three years, comparing these experiences with other studies that confirm the efficacy of liver transplantation not only in terms of survival but also of global quality of life improvement.

The interviewed patients reported high satisfaction levels in the post-transplant period. This indicates their ability to adapt to their new clinical condition, to process their care experience, and positively reinvest in their lives looking forward to their future with new confidence. The study also confirms the soundness of our model of global clinical and psychosocial care and continuous support to the transplant recipients.

## Figures and Tables

**Table 1:** Clinical and demographic characteristics.

*N =* 82		
Age, years	56 ± 9	
Male	62 (76%)	
Female	20 (24%)	
Main Etiology	HCC (33%)	
Transplant date	2017–2019	
MELD	Score 1	ISO > 30 = 11
	Score 2	20 < ISO < 30 = 53
	Score 3	ISO < 20 = 15

**Table 2:** Mean McGill Quality of Life Questionnaire© (MQOL) scales.

	Total Patients	Transplant Recipients Since over Two Years	Transplant Recipients Since Less than Two Years
		(*n* = 34)	(*n* = 46)
Total QOL	8/10	8	7
	(excellent)		
Physical well-being	8/10	8	7
	(excellent)		
Physical symptoms	24/30	25	24
	(no problem)		
Psychological symptoms	28/40	28	28
	(no problem)		
Existential well-being	49/60	51	46
	(excellent)		
Support	18/20	18	18
	(excellent)		

**Table 3:** Physical symptoms.

Main Physical Symptoms Reported	*N* Reported Cases
Abdominal hernia	7
Fatigue	6
Mucositis	1
Insomnia	1
Joint rigidity	2
Pain	3
Hcc recurrence	3

**Table 4:** Main studies on quality of life (QOL) and liver transplant.

Reference	Title	Study Design	Population (*n*)	Instruments Used to Assess QOL	Main Conclusions
Bravata, Olkin, Barnato, Keeffe, Owens (1999) [12]	Health-related quality of life after liver transplantation: a meta analysis	Meta-analysis	N/A	N/A	Transplant recipients reported large gains in those aspects of QOL most affected by physical health and smaller improvements in areas affected by psychological functioning.
Duffy, Kao, Ko, Farmer, McDiarmid, Hong et al. (2010) [13]	When does quality of life improve after liver transplantation? A longitudinal prospective study	Prospective, cross-sectional study	168	SF-36	More than 50% of LT recipients survive 20 years, achieve important socioeconomic milestones, and report quality of life superior to patients with liver disease or other chronic conditions.
Masala, Mannocci, Unim, Cimmuto, Turchetta, Gatto et al. (2012) [14]	Quality of life and physical activity in liver transplantation patients: Results of a case-control study in Italy	Case-control	45 transplant patients, 108 controls	SF-36	Transplant recipients are more subject to low physical functions and to psychological/emotional distress compared to the general population.
				IPAQ	
Wang, Yang, Li, Jiang, Fu, Jin et al. (2012) [15]	Health-related quality of life after liver transplantation: The experience from a single Chinese center	Case-control	60 post-LT, 55 benign end-stage liver disease, 50 controls	SF-36	In transplant recipients QOL remains to be improved, but generally they have a good QOL.
Sirivatanauksorn, Dumronggittigule, Limsrichamrern, Iramaneerat, Kolladarungkri, Kositamongkol et al. (2012) [16]	Quality of life among liver transplantation patients	Case-control	57 pre-LT 95 post-LT	SF-36	Liver transplant recipients and also their caregivers have a good QOL.
				CLDQ	
Ridolfi, Nanni Costa, Martinelli, Donati, Morselli Labate, Venturoli. [17]	Qualità di Vita ed Integrazione Sociale delle Persone Sottoposte a Trapianto dell’Organo Salvavita: Fegato	Cohort study	365	SF-36	QOL in transplant recipients returns to excellent levels, similarly to a control group population. The progressive improvement in time of the physical role and state score demonstrated that regaining social “normality” is in any case possible for most of these individuals.
				Q-Qsex	
Telles-Correia, Barbosa, Mega, Mateus, Monteiro (2009) [18]	When does quality of life improve after liver transplantation? A longitudinal prospective study	Cohort study	60	SF-36	QOL improved early after liver transplantation (1 month), mostly with a significant improvement in the physical quality of life.
Onghena, Develtere, Poppe, Geerts, Troisi, Vanlander (2016) [19]	Quality of life after liver transplantation: State of the art	Review	N/A	N/A	During the first year there is a significant improvement in QOL, which remains stable thereafter.
Painter, Krasnoff, Paul, Ascher (2001) [20]	Physical Activity and Health-Related Quality of Life in Liver Transplant Recipients	Cohort study	180	SF-36	This study indicates that physical activity is related to QOL after liver transplantation independent of other coexisting medical conditions.
Yang, Shan, Saxena, Morris (2014) [21]	Liver transplantation: a systematic review of long-term quality of life	Review	N/A	N/A	Liver transplantation confers specific long-term QOL and functional benefits when compared to preoperative status.
